# Electrochemical Detection Platform Based on RGO Functionalized with Diazonium Salt for DNA Hybridization

**DOI:** 10.3390/bios12010039

**Published:** 2022-01-13

**Authors:** Elena A. Chiticaru, Luisa Pilan, Mariana Ioniţă

**Affiliations:** 1 Faculty of Medical Engineering, University Politehnica of Bucharest, Gh Polizu 1-7, 011061 Bucharest, Romania; elenaa.chiticaru@yahoo.com; 2 Department of Inorganic Chemistry, Physical Chemistry and Electrochemistry, University Politehnica of Bucharest, Gh Polizu 1-7, 011061 Bucharest, Romania; 3 Advanced Polymer Materials Group, University Politehnica of Bucharest, Gh Polizu 1-7, 011061 Bucharest, Romania

**Keywords:** graphene, reduced graphene oxide, electrochemistry, electrochemical impedance spectroscopy, DNA biosensor, diazonium chemistry, screen-printed electrodes, DNA hybridization

## Abstract

In this paper, we propose an improved electrochemical platform based on graphene for the detection of DNA hybridization. Commercial screen-printed carbon electrodes (SPCEs) were used for this purpose due to their ease of functionalization and miniaturization opportunities. SPCEs were modified with reduced graphene oxide (RGO), offering a suitable surface for further functionalization. Therefore, aryl-carboxyl groups were integrated onto RGO-modified electrodes by electrochemical reduction of the corresponding diazonium salt to provide enough reaction sites for the covalent immobilization of amino-modified DNA probes. Our final goal was to determine the optimum conditions needed to fabricate a simple, label-free RGO-based electrochemical platform to detect the hybridization between two complementary single-stranded DNA molecules. Each modification step in the fabrication process was monitored by cyclic voltammetry (CV) and electrochemical impedance spectroscopy (EIS) using [Fe(CN)_6_]^3−/4−^ as a redox reporter. Although, the diazonium electrografted layer displayed the expected blocking effect of the charge transfer, the next steps in the modification procedure resulted in enhanced electron transfer properties of the electrode interface. We suggest that the improvement in the charge transfer after the DNA hybridization process could be exploited as a prospective sensing feature. The morphological and structural characterization of the modified electrodes performed by scanning electron microscopy (SEM) and Raman spectroscopy, respectively, were used to validate different modification steps in the platform fabrication process.

## 1. Introduction

Deoxyribonucleic acid (DNA) is a biopolymer that can self-assemble from two single-strands (ss) in a unique way that conforms to the Watson−Crick base pairing rules [1]. This process of specific self-assembly between two complementary polynucleotides is called hybridization and it is centered around the hydrogen bonds formed between the nucleobase base pairs, producing a double-helix structure [2]. The complementarity of DNA bases has been highly exploited in nanotechnology [3], in medicine [4] and in sensing applications [5,6]. The high specificity of the self-pairing between single-stranded DNA molecules can be used to detect specific sequences or biomarkers, which are related to bacterial [7] or viral [8] infections, for example. Moreover, the hybridization event can also be employed in the detection of DNA or messenger ribonucleic acid (mRNA) oligonucleotides, which can be extremely valuable in point-of-care applications [9], as well as for the diagnosis of various genetic mutations and diseases [10,11].

Electrochemical DNA biosensors are highly advantageous for the detection of particular ssDNA molecules due to their fast response time, inexpensive instrumentation and miniaturization potential [12]. Due to these advantages, DNA biosensors have been used so far in various applications, such as clinical diagnostics [13], drug interactions [14] and detection [15], and environmental monitoring [16]. The development of simple and effective electrochemical methods is of high interest in nucleic acid detection [17]. In this context, label-free approaches have attracted widespread interest as they can simplify the manufacturing processes and are free from the possible unfavorable effects of the labels, which proves their effectiveness for target sensing [18,19]. Many of these label-free detection strategies exploit the variation in the electrochemical signal of some charged freely diffusing redox species as a result of the change in their accessibility to the electrode surface driven by the target analyte binding [20]. One of the most employed redox species is the negatively charged [Fe(CN)_6_]^3−/4−^, and the hybridization events are detected by several methods, such as cyclic voltammetry (CV), differential pulse voltammetry (DPV), square wave voltammetry (SWV), and electrochemical impedance spectroscopy (EIS). 

The discovery of graphene and its related materials offers great opportunities for the design of superior electrochemical DNA biosensors due to their unique properties, such as high surface area, which can increase the immobilization capacity, increase the electron transfer rate, and can contribute to excellent biosensor sensitivity [21]. The functional groups present on the surface of graphene oxide (GO) can often be considered a disadvantage in sensing applications because they limit the electrical conductivity of the material. However, there are some strategies that exploit these functionalities by either removing them through different approaches (chemical, electrochemical, thermal, etc.) [22] or by increasing their reactivity through carbodiimide chemistry [23] in order to be further covalently linked to amino-terminated molecules. As pointed out in the recent literature, reduced graphene oxide (RGO) material offers a multitude of advantages compared to other biosensing transducer materials by providing a balance between the beneficial properties of pristine graphene and GO, and through its feasibility for various functionalization schemes suitable for the biorecognition elements immobilization [24,25]. It was confirmed by different studies [26,27,28] that graphene functionalization is substantially enhanced when the reaction is performed under electrochemical control. The Fermi level of graphene can be shifted by adjusting the applied electrochemical potential, which is a simpler way to increase the reactivity of the material than using aggressive chemicals to disrupt the covalent sp^2^ bonds [29]. Among numerous functionalization strategies, the electrochemical reduction of diazonium salts [30,31,32,33,34] has been proposed as a versatile method that affords the grafting of a large variety of molecules at conductive substrates [35,36]. So far it has been applied for different types of carbon-based substrates such as glassy carbon [37], screen-printed carbon [38], carbon paste [39], graphite pencil [40] or carbon nanomaterials electrodes [41,42], that function as transducers in a large number of sensors for bioanalytes, such as proteins [43], DNA [44], RNA or cells [45]. Although great effort has been dedicated to this subject, there is still no refined method available to mass-produce highly stable, efficient biosensors with real applicability in biomedicine. Therefore, our aim was to design an improved DNA detection platform in terms of stability, sensitivity and reproducibility, by modulating the electrochemical parameters.

In a previous study [25], we have developed an electrochemical platform for DNA detection by physical adsorption of DNA probe (DNAp) molecules onto RGO-modified electrodes. Moreover, we have introduced a new protocol for the pretreatment of screen-printed carbon electrodes (SPCEs) that were used in our work, which improved their electrochemical properties. Considering that the immobilization of oligonucleotides through physical adsorption does not permit a high level of precision and control for DNA attachment on the substrate, we are proposing a new approach that consists in the covalent immobilization of amino-modified DNA probes onto a carboxylic functionalized RGO surface. This strategy provides stronger and highly specific binding between the bioreceptor and the working electrode by eliminating the possibility of DNA desorption [12], as opposed to the non-covalent method [46,47]. The amino-modified ssDNA probes are covalently attached at the RGO substrate through stable amide link via carbodiimide coupling reaction. Unlike other literature studies that report the same coupling chemistry for the biorecognition element at the carboxylic functions of the GO [48], this work also exploits the significant advantages of diazonium chemistry to provide the stable and controlled functionalization of the RGO electrode with aryl carboxylic groups (Ar-COOH) [46], with the implied benefits for ssDNA probe immobilization. A reproducible platform for DNA hybridization detection was envisaged by putting special emphasis on the electrochemically controlled fabrication steps for both the preparation of the RGO substrate electrode and the covalent binding of the biorecognition element. In addition to the portability and cost-effectiveness of SPCEs, the reproducible modification of these substrates with RGO and the controlled covalent binding of the biorecognition element can enable the fabrication of competitive, stable, reproducible and novel platforms for DNA hybridization detection. The effect of the successive steps in the sensor fabrication process was established by the electrochemical properties of the modified electrodes quantified by the charge transfer resistance (Rct) and the electron transfer rate constant (k^0^) parameters. The electrochemical results were in agreement with morphological and structural characterization studies performed by scanning electron microscopy (SEM) and Raman spectroscopy, respectively. The improvement in the charge transfer after the DNA hybridization process could be exploited as a prospective sensing feature.

## 2. Materials and Methods

### 2.1. Reagents and Materials

Graphene oxide dispersion in water (2 mg/mL), KCl, HCl, H_2_NaO_4_P, HNa_2_O_4_P, N-(3-Dimethylaminopropyl)-N′-ethylcarbodiimide hydrochloride (EDC), N-Hydroxysulfosuccinimide sodium salt (NHS), NaNO_2_ and 4-aminobenzoic acid (4-ABA) were supplied by Sigma-Aldrich (St. Louis, MO, USA). Potassium ferricyanide (K_3_[Fe(CN)_6_]) and potassium ferrocyanide (K_4_[Fe(CN)_6_] × 3H_2_O) were purchased from Merck Co., (Darmstadt, Germany). Single-stranded DNA probe modified with amino groups (5′-AmC6-TTT CAA CAT CAG TCT GAT AAG CTA TCT CCC-3′), single-stranded DNA target (DNAt, 5′-GGG AGA TAG CTT ATC AGA CTG ATG TTG AAA-3′) and 10 mM Tris, 0.1 mM EDTA (IDTE) buffer were acquired from Integrated DNA Technologies, Inc (Coralville, IA, USA). The SPCEs were thoroughly rinsed with ultrapure water (Adrona Crystal EX water purification system, 18.2 MΩ × cm resistivity) after each modification. Screen-printed carbon electrodes (SPCE-DRP 110) were purchased from Metrohm DropSens, Spain.

### 2.2. Procedures

#### 2.2.1. Electrochemical Measurements

The SPCEs consisted of a 4 mm diameter working electrode (WE), a silver pseudo-reference electrode (RE), and a carbon counter electrode (CE). Each SPCE modification was investigated by cyclic voltammetry and electrochemical impedance spectroscopy, which were performed at room temperature using a potentiostat/galvanostat Autolab PGSTAT 204 (Metrohm Autolab, Netherlands) equipped with NOVA 2.1 software. The SPCE was attached to a connector (DSC) device from Metrohm Dropsens, functioning as an interface between the SPCE and the potentiostat. The electrochemical measurements were recorded by adding 100 µL 0.1 M KCl electrolyte solution containing 1 mM [Fe(CN)_6_]^3−/4−^ redox probe on the SPCE. CV curves were recorded by scanning the potential between −0.2 V and +0.6 V, at a sweep of 0.05 V/s (unless stated otherwise). The impedance spectra were recorded in the frequency range of 0.01–10^5^ Hz, with 10 mV AC amplitude at an applied potential of 0.16 V, which is the formal potential of the [Fe(CN)_6_]^4−/3−^ redox probe vs. the Ag pseudo reference electrode.

#### 2.2.2. Morphological and Structural Characterization

A FEI high-resolution focused ion beam scanning electron microscopy (FIB-SEM) system model Versa 3D DualBeam (FEI Company, Hillsboro, OR, USA) was used to characterize the selected sample series. The plane view (0° tilt) samples’ surface morphology were investigated by detecting the secondary electrons (SE) signals in High-Vacuum operation mode (6.1 × 10^−4^ Pa) at a working distance of 10 mm, using 10 kV as the accelerating voltage and a spot size of 4.5. Moreover, the SmartSCAN scanning strategy and DCFI drift suppression features of Versa 3D DualBeam tool were involved to fully ensure the imaging stability.

Raman spectroscopy was performed in order to investigate the structural changes on the surface of the electrodes. Raman spectra were obtained with a Renishaw inVia Raman confocal spectrometer (Renishaw, Brno-Černovic, Czech Republic), using a laser excitation wavelength of 473 nm and the 100× objective.

#### 2.2.3. RGO-Modified Electrode Preparation Procedure

The electrodes coated with electrochemically reduced graphene oxide (further denoted as RGO/SCPE) were prepared following the procedure described in [25]. Before any modification, the SPCEs were pretreated by 5 voltametric cycles performed in 0.1 M HCl, from +0.5 to −1.5 V, at the scan rate of 0.05 V/s, followed by 5 cycles in 0.1 M phosphate buffer solution (PBS), pH 7, from 0 to +2 V, 0.05 V/s. This activation treatment ensures a higher electron transfer rate for the redox probe at SPCE, illustrated by a smaller peak to peak separation in the CV signal [25]. Subsequently, prior to the graphene modification step, 3 µL PBS was deposited on the WE surface, and then the electrodes were washed with ultrapure water and dried at 60 °C. As mentioned previously [25], this step is highly important because it changes the surface properties of the carbon surface and it guarantees a reproducible GO modification. Once the SPCEs cooled down, 3 µL of GO dispersion (0.3 mg/mL) was cast on their surface, then dried at 60 °C for 2 h and kept at room temperature overnight. Finally, the GO/SPCEs were reduced electrochemically by 10 CV cycles applying a potential between 0 and −1.5 V, at a scan rate of 0.1 V/s, in 0.5 M KCl. The measurements were performed in triplicate in order to ensure the reproducibility of the procedure.

#### 2.2.4. RGO/SPCE Functionalization by Diazonium Chemistry

The functionalization of RGO/SPCEs with carboxyphenyl layer was conducted with in situ generated aryldiazonium cations that involved the electrochemical reduction of the corresponding aniline (4-aminobenzoic acid) in acidic media. Specifically, 2 mM 4-aminobenzoic acid was solubilized in 0.5 M aqueous HCl, to which 2 mM of sodium nitrite was added to generate the aryl diazonium salt. After 5 min stirring at room temperature, 100 µL of mixture was immediately added onto the electrode surface, so as to cover all connections. The electrochemical procedure employed for grafting was based on the protocol described by Eissa et al. [49] and consisted in potential cycling for one cycle between +0.3 V and −0.6 V, with a scan rate of 0.1 V/s. At this stage, an irreversible reduction peak should be observed as a preliminary validation of the functionalization process. After surface derivatization, the electrodes were rinsed with high amounts of ultrapure water. The blocking properties of the modified electrodes evaluated by CV and EIS in the presence of soluble electroactive [Fe(CN)_6_]^3−/4−^ species also confirmed this grafting step.

#### 2.2.5. Fabrication of DNA Biosensor

The DNA biosensor was fabricated by covalently immobilizing the amino ssDNA probe onto RGO/SPCE using carbodiimide chemistry. For this purpose, 0.2 M EDC and 0.05 M NHS in 0.1 M PBS buffer solution of pH 6, were used as activation agents for the carboxyl groups confined at the electrode surface. After 2 h activation at room temperature, the Ar-COOH/RGO/SPCEs were immersed overnight in the ssDNA probe solutions at room temperature to obtain the detecting electrodes. Fresh DNA probes were prepared from the stock solution of 100 µM to 10, 5, 1, and 0.5 µM using IDTE buffer to dilute. The hybridization testing of the developed platform for the complementary target DNA sequence was achieved by dropping an appropriate concentration of target DNA solution (in IDTE buffer) onto the SPCE modified with the recognition layer and incubating at room temperature for 2 h. For longer times (i.e., 4 h and 6 h), no significant change in the electrochemical studies occurred, indicating that the ssDNA probes immobilized onto SPCE were completely hybridized by the target DNA after 2 h.

## 3. Results and Discussions

### 3.1. Morphological Characterization

**Figure 1:**
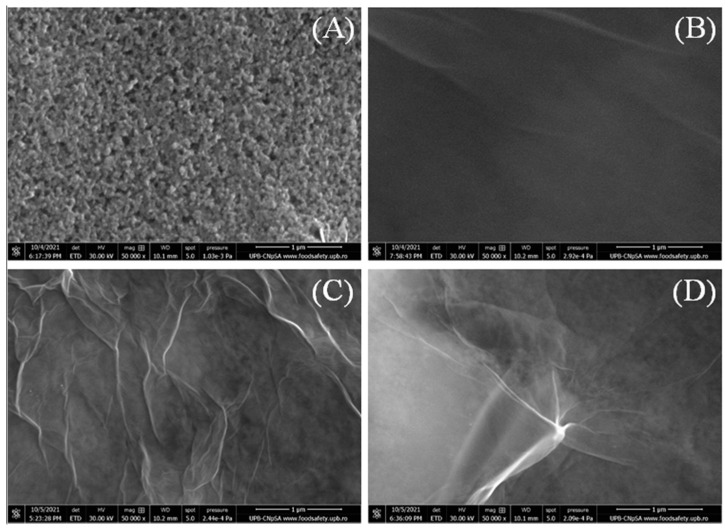
SEM images of bare SPCE (**A**), GO/SPCE (**B**), RGO/SCPE (**C**), and Ar-COOH/RGO/SPCE (**D**). Images recorded at 50 kX magnification.

The morphology of the modified electrodes was investigated by SEM, which is also a useful technique to determine if the surface of the working electrodes is uniformly covered with graphenic material. Several images were acquired from different spots on the working electrodes and the most representative are presented in Figure 1. First of all, the bare SPCE (Figure 1A) was studied and it revealed a rough porous surface similar to the one previously obtained by us [25]. The image corresponding to GO/SPCE (Figure 1B) shows homogenous coverage of the electrode surface with thin, well-dispersed graphene oxide sheets, while RGO deposited on SPCE (Figure 1C) displays the typical graphene morphology with wrinkled layers uniformly covering the whole working electrode. Finally, Ar-COOH/RGO/SPCE (Figure 1D) reveals a morphology similar to the other electrodes coated with graphenic species, showing thin graphene layers with creased margins. All in all, the SEM images emphasize the flexibility of the graphenic sheets and confirm the homogenous coverage of the electrodes with a thin layer of GO dispersion, while the porous morphology of the carbon electrode can still be observed underneath.

### 3.2. Structural Characterization

**Figure 2:**
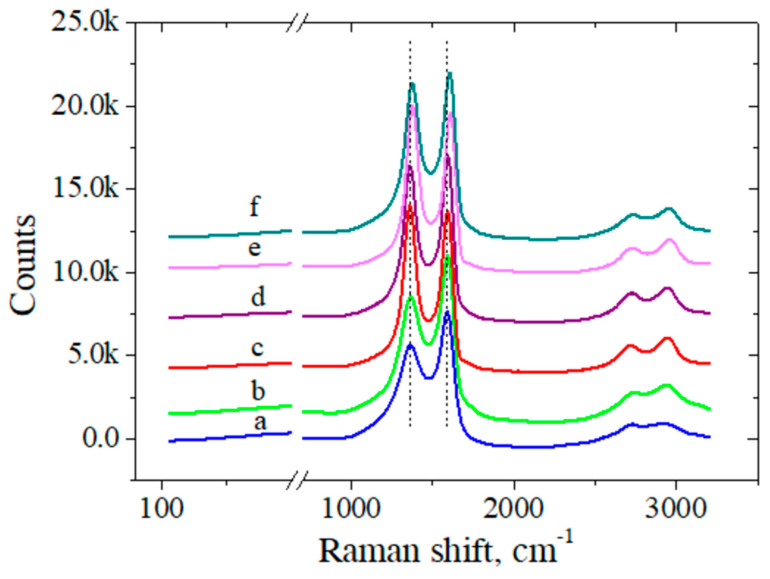
Raman spectra of bare SPCE (**a**), GO/SPCE (**b**), RGO/SCPE (**c**), Ar–COOH/RGO/SPCE (**d**), Ar–COOH/RGO/SPCE activated in EDC–NHS (**e**), and ssDNAp/Ar–COOH/RGO/SPCE (**f**).

Raman spectra (Figure 2) were recorded after each step of electrode modification to investigate the changes in the graphenic structure. I_D_/I_G_ ratio is higher for RGO compared to GO due to the many structural defects that appeared as a consequence of removing functional groups from the GO surface. Once Ar-COOH groups were grafted on the electrode surface, more sp^2^ carbon bonds were formed and the G peak increased more than the D band. The I_D_/I_G_ ratio increased again after activation in EDC-NHS and decreased after electrode incubation in the ssDNA probe, confirming the immobilization of oligonucleotides on the activated Ar-COOH/RGO/SPCE platform. Moreover, the position of D and G vibrational bands was at approximately 1360 cm^−1^ and 1590 cm^−1^, respectively, which is characteristic of graphene and its derivatives [50]. These two peaks change their position after each electrode modification, recording the biggest shift after ssDNA probe immobilization, which strongly suggests that the bioreceptor is indeed bound to the electrode surface. The positions of the D and G bands along with the I_D_/I_G_ ratios are presented in Table 1.

### 3.3. Electrochemical Characterization

#### 3.3.1. Carboxyphenyl Electrografted RGO Electrodes

**Figure 3:**
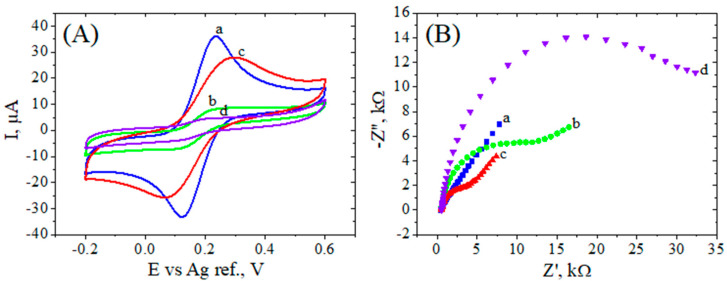
Electrochemical characterization for bare SPCE (**a**), GO/SPCE (**b**), RGO/SPCE (**c**), and Ar–COOH/RGO/SPCE (**d**) modified electrodes: CVs, 0.05 V/s (**A**) and EIS Nyquist plots at 0.16 V vs. Ag ref. (**B**) recorded in 1 mM [Fe(CN)_6_]^3−/4−^, 0.1 M KCl.

The modification of commercial electrodes with RGO by applying our former optimized protocol [25] with minor changes has been described in Section 2. As presented in Figure 3, the modification of SPCEs with GO caused a decrease in the current peaks that correlated with a high charge transfer resistance, characteristic to the poor conductive properties of the oxygenated graphene. Nevertheless, the conductivity was improved with the electrochemical reduction of GO, as can be seen by a substantial increase in the redox peaks and a decrease in R_ct_. The subsequent diazonium electrografting of the electrode surface with 4-carboxyphenyl moieties induced a significant decrease in peak currents attributed to the electrostatic repulsion of negatively charged COOH groups (at neutral pH) for the [Fe(CN)_6_]^3−/4−^ ions (Figure 3A). The Nyquist plot of the carboxylic-grafted RGO displayed a semicircle for the whole frequency domain, indicating that the interfacial charge transfer dominates over mass transport effects (Figure 3B). The modification of SPCE with GO dispersion was also confirmed by SEM analysis, which displayed a change in the WE morphology (see Section 3.1), while the changes in the electrochemical properties observed after each SPCE modification were well correlated with the variations in the I_D_/I_G_ ratios from the Raman spectra reported in Section 3.2.

The typical mechanism for graphene covalent functionalization with aryl diazonium salts consists in the transport of a delocalized electron from the graphene lattice to the aryl diazonium cation that releases a nitrogen molecule and leads to a highly reactive aryl radical [51]. These radicals then form covalent bonds with the carbon atoms of the graphene structure, causing the conversion of the sp^2^-carbon in the graphene sheet to sp^3^ hybridization [52]. Although there are many challenges related to the precise control of the structure of the aryl layers, the use of diazonium chemistry offers advantages in terms of simplicity and long-term stability of the biorecognition layer [53]. Moreover, modulating the degree of functionalization by using the electrochemical route for the reduction of aryl diazonium salts represents an efficient alternative to traditional methods for graphene functionalization [54].

**Figure 4:**
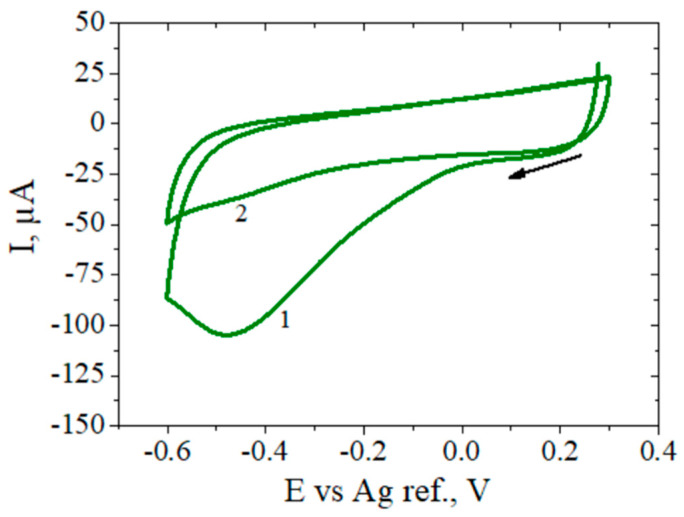
CVs corresponding to the 1st and 2nd cycles of the reduction of 4–carboxyphenyl diazonium salt (generated in situ) at the RGO/SPCE (potential sweep from +0.3 V to −0.6 V at 0.1 V/s).

The functionalization of the RGO surface was performed by electrochemical reduction of in situ generated carboxyphenyl diazonium salt in acidic aqueous solution by CV, from 0.3 to −0.6 V vs. Ag ref. with a scan rate of 0.1 V/s. Upon first scanning, an irreversible reduction wave was observed at −0.5 V vs. Ag ref., which can be attributed to the formation of the 4-carboxyphenyl radicals with subsequent covalent bond formation with the graphene surface. In a second performed cycle, the cathodic peak disappears, indicating the coverage of the RGO surface with the carboxyphenyl groups that block further electron transfer between the diazonium cations in the solution and the modified electrode (Figure 4). This behavior agrees well with previously reported studies on the reduction of diazonium cations onto other carbon materials in various forms [33,36]. These results demonstrated that one reduction cycle is sufficient to saturate the electrode surface with functional carboxyphenyl groups, and in the biosensor fabrication process we employed one CV cycle at 0.1 V/s, which ensured a reproducible electrode. Moreover, extended grafting was avoided in order to prevent the formation of poorly conducting multilayers, which can create a significant barrier to electron transfer between the electrode and the redox species in the solution [55,56].

#### 3.3.2. Amino-Modified ssDNA Probe Immobilization

**Figure 5:**
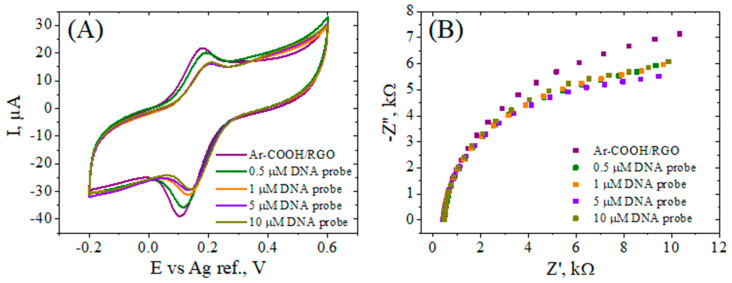
CV (**A**) and EIS Nyquist plot (**B**) recorded in 1 mM [Fe(CN)_6_]^3−/4−^, 0.1 M KCl, for Ar–COOH/RGO/SPCE activated in EDC–NHS, and incubated with 0.5, 1, 5, and 10 µM amino–modified ssDNA probe.

The amine-modified ssDNA probe was covalently attached to the carboxyl groups of the aryl layer through carbodiimide coupling, in the presence of EDC and NHS as activating agents. In order to determine a saturation level, different concentrations for the ssDNA solution in IDTE buffer (0.5, 1, 5, and 10 µM) were tested for the coupling reaction. As depicted in Figure 5A, the CV tests for the ssDNA-modified electrodes in the presence of [Fe(CN)_6_]^3−/4−^ redox probes revealed consecutive losses in the peak currents’ intensity with an increasing concentration up to 5–10 μM, at which values, the response signal stagnated. Based on these results, we considered that at 10 µM the electrode surface was fully covered with specifically linked oligonucleotides, and this concentration of ssDNA probe was chosen to further check the hybridization process. Interestingly, this decrease in the peak currents was accompanied by a decrease in the peaks’ potential separation (ΔE_p_). The ΔE_p_ trend in the CV response was sustained by the results obtained by faradaic impedance measurements (Figure 5B), with a decrease in R_ct_ upon ssDNA probe immobilization, which suggests an improvement in the kinetics of the [Fe(CN)_6_]^3−/4−^ redox species at the ssDNA-modified electrodes. This decrease in the R_ct_, despite the presence of the negatively-charged DNA backbone, which is prone to repulse the ferri/ferrocyanide ion, can be explained by an improvement in the hydrophilicity [57] of the interface in comparison with the electrode simply modified with the aryl grafted layer.

#### 3.3.3. The Sensor Response for DNA Target Molecule

**Figure 6:**
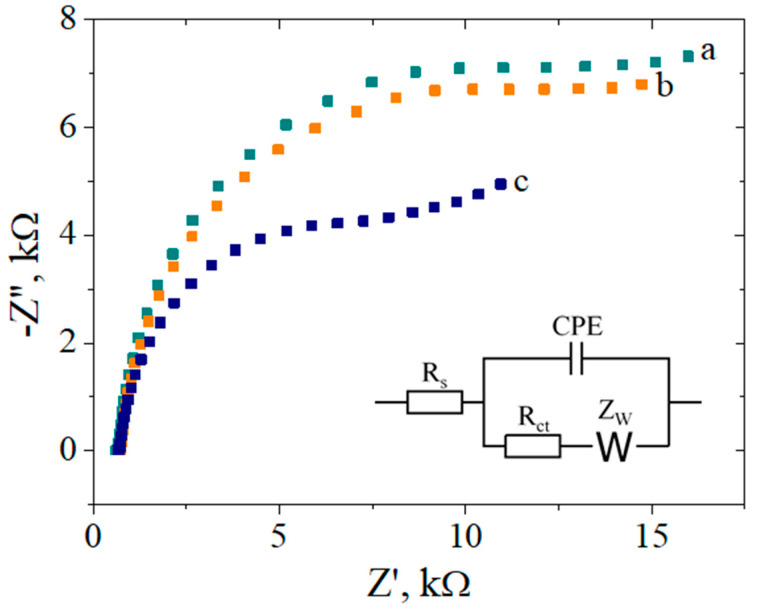
EIS Nyquist plot recorded in 1 mM [Fe(CN)_6_]^3−/4−^, 0.1 M KCl, for the Ar–COOH/RGO–modified electrodes after activation of the carboxylic groups (**a**), after carbodiimide coupling with 10 µM NH_2_–DNA probe (**b**) and after hybridization with 200 nM DNA target (**c**). Inset: Randles equivalent circuit used for fitting.

The hybridization between the probe and target DNA was monitored by using the same ferri/ferrocyanide mixture as a redox indicator, while the EIS technique was employed to evaluate the recognition event (Figure 6). Table 2 presents the charge transfer resistance values for the different steps in the electrode modification, obtained by fitting the Nyquist spectra using a Randles equivalent circuit (inset Figure 6). Modification of the RGO electrode with Ar-COOH groups induced a large increase in the R_ct_ value due to the electrostatic repulsion between the redox probe and the negatively charged carboxylic groups. Still, as a result of the activation reaction with EDC/NHS and the formation of the NHS ester, a significant decrease in its value was then observed. Although single-stranded DNA probe molecules are also negatively charged and prone to repulse the ferri/ferrocyanide ions, the further slight decrease in R_ct_ might be explained by an increase in the hydrophilic character of DNA functionalized electrodes compared with Ar-COOH/RGO/SPCE, which aids the access of the negative redox couple to the electrode surface [44].

The spectrum (c) in Figure 6 shows a significant decrease in R_ct_ upon DNA target binding, which constitutes the basis of the detection process. This significant decrease in charge-transfer resistance during hybridization can be associated with the morphological changes resulting from the formation of the “rigid rod” double stranded (ds) DNA. Similar behavior has been observed by Gooding et al. [58] for the free electrochemical detection of DNA hybridization at SAM-modified Au electrodes. According to their studies, the hybridization opens the interface, allowing redox species in the solution to access the surface and the electrochemical reaction to occur. This atypical decrease in R_ct_ can be justified considering that ssDNA probes behave as flexible molecules that lie down across the modified electrode, thus contributing with direct electrostatic repulsion to the negatively charged redox probe. Conversely, the more rigid dsDNA structure generated after the hybridization process provides increased access of the [Fe(CN)_6_]^3−/4−^ redox probe to the electrode surface, and thus, improved electron transfer. This significant change in R_ct_ following DNA hybridization suggests that this electrochemical platform has great potential as a high sensitivity DNA biosensor.

#### 3.3.4. Assessment of the Electron Transfer Kinetics at the RGO-Modified Electrodes

**Figure 7:**
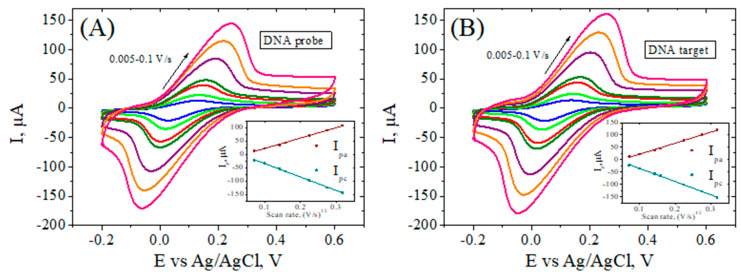
CV curves of ssDNAp/Ar–COOH/RGO/SPCE (**A**), and ssDNAp/Ar–COOH/RGO/SPCE after hybridization with DNA target (**B**) recorded at different scan rates in 1 mM [Fe(CN)_6_]^3−/4−^, 0.1 M KCl solution. Inset: plot of anodic and cathodic peaks vs. square root of scan rate.

In order to compare the charge transfer properties of the modified electrodes, we further evaluated heterogeneous electron transfer rate constants for the [Fe(CN)_6_]^3−/4−^ redox couple. Scan dependence CV studies performed at RGO/SPCE, Ar-COOH/RGO/SPCE, ssDNAp/Ar-COOH/RGO/SPCE, and DNAt/ssDNAp/Ar-COOH/RGO/SPCE (Figure 7) displayed an increase in the peak-to-peak separation potential for all electrodes except Ar-COOH/RGO/SPCE (not shown), for which passivation occurs due to grafted carboxylic layers that suppress further electrochemistry of Fe(CN)_6_^3−/4−^ species. In all other cases, anodic and cathodic peak currents increase with increasing potential scan rate and are linearly related to the square root of the scan rates in the range of 0.005–0.1 V/s (see the insets in Figure 7), suggesting that the oxido-reduction process at the electrode surface is controlled by diffusion. The Nicholson methodology [59] was applied to estimate k^0^ for the quasi-reversible process of [Fe(CN)_6_]^3−/4−^ at the modified electrodes. The values of the diffusion coefficients of the oxidized and reduced forms of [Fe(CN)_6_]^3−/4−^ D_O_ = 7.6 × 10^−6^ cm^2^ s^−1^, D_R_ = 6.5 × 10^−6^ cm^2^ s^−1^ were taken from the literature [60], and the dimensionless kinetic parameter Ψ was calculated by using the equation given by Lavagnini et al. [61]. The calculated k^0^ values for the Ar-COOH/RGO/SPCE-modified electrodes after the ssDNA probe immobilization, and the hybridization with DNA target are 1.65 × 10^−3^ cm s^−1^, and 2.06 × 10^−3^ cm s^−1^, respectively. This trend is in agreement with the EIS results reported before, showing an improvement in the charge transfer after DNA hybridization process.

**Figure 8:**
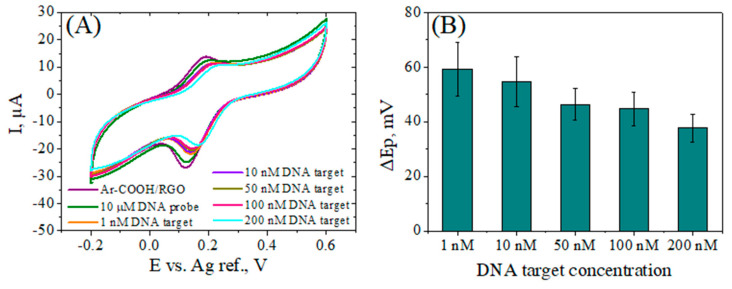
CV curves (**A**) and the corresponding peak separation values, ΔEp (**B**) of ssDNAp/Ar–COOH/RGO/SPCE after hybridization with different concentration of DNA target, recorded at a scan rate of 0.05 Vs^−1^ in 1 mM [Fe(CN)_6_]^3−/4−^, 0.1 M KCl solution.

The same pattern in the sensor response was observed throughout the concentration range of 1–200 nM, i.e., a smaller peak separation by DNA target addition, suggesting an increase in the charge transfer rate with target concentration (Figure 8). These evident changes in heterogeneous electron transfer as a result of the hybridization process should support the further use of this detection platform for the advancement of DNA biosensors.

## 4. Conclusions

In summary, we have developed an RGO-based stable and reproducible biosensing platform capable of achieving label-free and sensitive detection of DNA hybridization. To the best of our knowledge, no attempts have been presented to date in the literature to develop electrochemical label-free DNA detection platforms at graphene electrodes by ssDNA probe immobilization through diazonium chemistry. The diazonium-grafted layers allow for the controlled immobilization of oligonucleotide probes at the RGO substrate, which results in stability and hybridization efficiency. Our label-free diazonium-based strategy relies on the changes in the accessibility of a redox probe in the solution to the RGO electrode surface, as a consequence of the changes in the flexibility of the DNA oligonucleotides upon hybridization. This conclusion was supported by the kinetic study performed at the RGO-modified electrodes with ssDNA probes and in the presence of target, which demonstrated a faster redox reaction after the hybridization process as the resulting rigid dsDNA structure facilitates the redox probe access to the electrode surface. In addition, the modification of SPCE with GO dispersion was evidenced by SEM analysis, while the electrochemical features observed by CV and EIS after each electrode modification were in agreement with the variations in the I_D_/I_G_ ratio from the Raman spectra. This new, simple, reproducible protocol proposed by us has great potential to be employed for the future manufacturing of miniaturized, sensitive and stable DNA biosensors.

## Figures and Tables

**Table 1:** Positions of D and G bands and their I_D_/I_G_ ratio from Raman spectra.

Electrode Modification	D Band [cm^−1^]	G Band [cm^−1^]	I_D_/I_G_ Ratio
SPCE	1362.9	1589.4	0.7167
GO/SPCE	1363	1591.4	0.7739
RGO/SPCE	1359.9	1591.7	1.0228
Ar–COOH/RGO/SPCE	1360.9	1593.6	0.9647
Ar–COOH/RGO/SPCE activated	1375.3	1608.3	1.0232
ssDNAp/Ar–COOH/RGO/SPCE	1373.6	1604	0.9713

**Table 2:** Charge transfer resistance values obtained from EIS measurements for the different stages in the electrode modification, along with the quality of fitting χ^2^ and standard deviation (SD).

Electrode Modification	Rct	χ^2^	SD
GO	10.5	0.0149	0.2121
RGO	1.7	0.0143	0.3111
Ar–COOH/RGO	36.3	0.0295	9.3338
Ar–COOH/RGO activated	13.5	0.0367	0.2828
ssDNAp/Ar–COOH/RGO	11.5	0.0236	0.9192
DNAt/ssDNAp/Ar–COOH/RGO	7.4	0.0178	0.7212
